# Structural Variations of the 3D Genome Architecture in Cervical Cancer Development

**DOI:** 10.3389/fcell.2021.706375

**Published:** 2021-07-23

**Authors:** Muhammad Muzammal Adeel, Hao Jiang, Yibeltal Arega, Kai Cao, Da Lin, Canhui Cao, Gang Cao, Peng Wu, Guoliang Li

**Affiliations:** ^1^National Key Laboratory of Crop Genetic Improvement, Huazhong Agricultural University, Wuhan, China; ^2^Agricultural Bioinformatics Key Laboratory of Hubei Province, Hubei Engineering Technology Research Center of Agricultural Big Data, 3D Genomics Research Center, College of Informatics, Huazhong Agricultural University, Wuhan, China; ^3^State Key Laboratory of Agricultural Microbiology, Huazhong Agricultural University, Wuhan, China; ^4^College of Veterinary Medicine, Huazhong Agricultural University, Wuhan, China; ^5^College of Bio-Medicine and Health, Huazhong Agricultural University, Wuhan, China; ^6^Department of Gynecologic Oncology, Tongji Hospital, Tongji Medical College, Huazhong University of Science and Technology, Wuhan, China; ^7^Cancer Biology Research Center (Key Laboratory of the Ministry of Education), Tongji Hospital, Tongji Medical College, Huazhong University of Science and Technology, Wuhan, China

**Keywords:** cervical cancer, gene expression, Hi-C, SVs, translocation detection, topologically associating domains

## Abstract

Human papillomavirus (HPV) integration is the major contributor to cervical cancer (CC) development by inducing structural variations (SVs) in the human genome. SVs are directly associated with the three-dimensional (3D) genome structure leading to cancer development. The detection of SVs is not a trivial task, and several genome-wide techniques have greatly helped in the identification of SVs in the cancerous genome. However, in cervical cancer, precise prediction of SVs mainly translocations and their effects on 3D-genome and gene expression still need to be explored. Here, we have used high-throughput chromosome conformation capture (Hi-C) data of cervical cancer to detect the SVs, especially the translocations, and validated it through whole-genome sequencing (WGS) data. We found that the cervical cancer 3D-genome architecture rearranges itself as compared to that in the normal tissue, and 24% of the total genome switches their A/B compartments. Moreover, translocation detection from Hi-C data showed the presence of high-resolution *t*(4;7) (q13.1; q31.32) and *t*(1;16) (q21.2; q22.1) translocations, which disrupted the expression of the genes located at and nearby positions. Enrichment analysis suggested that the disrupted genes were mainly involved in controlling cervical cancer-related pathways. In summary, we detect the novel SVs through Hi-C data and unfold the association among genome-reorganization, translocations, and gene expression regulation. The results help understand the underlying pathogenicity mechanism of SVs in cervical cancer development and identify the targeted therapeutics against cervical cancer.

## Introduction

Cervical cancer (CC) is the fourth most common cancer affecting women worldwide. With an estimated 570,000 cases and 311,000 deaths in 2018, this disease accounts for 3.3% of all cancer-related deaths (Bray et al., 2018), and there is a wide variation in incidence and mortality in various regions. In general, cancer is characterized by uncontrolled growth and cell proliferation due to several genomic changes such as gene mutations, insertion/deletions, and chromosomal rearrangements (Engreitz et al., 2012). In China, oncogenic HPV infection in women has been reported as 5–20%, depending on location and age (Münger et al., 2004; Dai et al., 2006; Zhao et al., 2012). Several studies have suggested that human papillomavirus (HPV) is the leading cause of cervical cancer and HPV genome integration is the key mechanism. Previously reported studies had suggested that the HPV integration hotspots, molecular pathogenesis, the role of episomal HPV E6/E7 expression, and HPV integration in human genome 3D structure (Fudenberg et al., 2011; Koneva et al., 2018; Cao et al., 2020) play a vital role in cervical cancer development (Garraway and Lander, 2013).

Structural variations (SVs) such as deletions, duplications, insertion, inversions, and translocations are majorly associated with disease development. Chromosome conformation capture techniques such as Hi-C and ChIA-PET have revealed that SVs alter the three-dimensional (3D) genome and gene regulations in the cancer genome (Dixon et al., 2018). SVs, specifically translocations that occur at specific hotspots in the genome, cause a significant impact on the 3D structure and gene expression (Lawrence et al., 2013). The detection of SVs and their effects on chromosomal architecture and gene expression has significantly increased our understandings of tumor development (Dixon et al., 2018). Multiple conventional techniques such as Microarray (Alkan et al., 2011), fluorescence *in situ* hybridization (FISH) (Cui et al., 2016), and PCR are already available to identify SVs (Sanchis-Juan et al., 2018). However, these methods have some drawbacks because they required prior knowledge (Mardis and Wilson, 2009); most of the techniques cannot accurately locate the sequence of breakpoints, making it more challenging to monitor the impact of specific SVs on gene structure (Hu et al., 2020). Nowadays, several studies have been designed to apply the most advanced high-throughput techniques such as whole-genome sequencing (WGS), RNA-seq, and chromosome conformation capture (Hi-C) data to study the SVs effectively (Dixon et al., 2018).

Despite the massive ongoing progress in cancer studies, there is still plenty of room to devise comprehensive research that uses an integrative approach to study SVs and their consequences in the cervical cancer genome.

Here, we have used normal and cervical cancer tissue data high-throughput chromosome conformation capture (Hi-C), transcriptome (RNA-seq), and WGS to identify SVs, specifically translocations. We monitored their local and global effects on the chromosomal 3D organization and gene expression. The results will help us to get a better insight into the correlation between SVs, specifically translocations and expression of oncogenes in cervical cancer.

## Materials and Methods

### Data Source

Hi-C data generated from Digestion Ligation Only Hi-C (DLO Hi-C) technique (Lin et al., 2018), WGS, and RNA-sequence data for normal and cervical cancer tissues were downloaded from Genome Sequence Archives^1^ under accession number CRA001401.

### Hi-C Data Processing and Breakpoint Detection

For Hi-C data processing, we used human genome hg19 and HPV-16 genome merged assembly as a reference genome. First, quality control of raw fastq files was performed with FastQC v0.11.8 (Andrews, 2010). The DLO Hi-C tool (Hong et al., 2019) was used to process the Hi-C data generated by the Digestion-ligation-only Hi-C technique (Lin et al., 2018). This tool removes pair end tags (PETs) of self-ligation, re-ligation, and dangling pairs. The contact matrices at different resolutions were normalized using ICE method (Imakaev et al., 2012). Topologically associated domains (TADs) and TAD boundaries at 40 kb resolution were identified using TopDom R-Package at default parameters (Shin et al., 2016). Juicer eigenvector was used to define A/B compartment, and bins with positive values were considered as A compartments, while bins with negative values were defined as B compartment at 500 kb resolution (Durand et al., 2016b). HiTC Bioconductor Package was used for quality control analysis of Hi-C data (Servant et al., 2012).

As we know, Hi-C data represent the contact probabilities between two regions of interacting chromosomes in a matrix form, which enables the detection of translocation. So, we used publically available pipelines such as HiCtrans (Chakraborty and Ay, 2018) and hic_breakfinder^2^, which use Hi-C data to find translocations. HiCTrans takes Hi-C contact matrices as an input to find translocation breakpoints based on change point values obtained by calculating the contact probability across each chromosomal contact pair (Chakraborty and Ay, 2018). hic_breakfinder uses mapped file (^∗^.bam file) as an input and human genome assembly-based filtering list of false positives and reports refined translocations at different resolutions (1 Mb, 100 kb, and 10 kb). Moreover, we used an *in-house* build script that uses a valid-pairs file of Hi-C data in *bedpe* format and detects chromosomal breakpoints. The resulted breakpoints of all tools were compared by using *bedtools pairToPair* to find overlapped and unique translocated regions.

### Whole-Genome Sequence Data Analysis and Structural Variation Detection

After quality control check of cervical cancer tissue and normal blood WGS data (Experiment ID: CRX040585 and CRX101064), through FastQC (Andrews, 2010) and Trimmomatic (Bolger et al., 2014), refined raw reads were aligned against proxy genome (hg19 + HPV16) using Burrows–Wheeler Alignment (BWA) tool (Li and Durbin, 2009) at default parameters, and the duplicates were marked and removed using Picard^3^. SAMtools was used for alignment quality estimation and sorting bam reads (Li et al., 2009). For SV detection in cervical cancer tissue WGS data (Experiment ID: CRX040585), we used Manta-tumor only (Chen et al., 2016). SV caller at default settings, additional refinement “PASS” parameter was applied, and results were visualized by Integrative Genome Viewer (IGV) (Robinson et al., 2011). ANNOVAR was used to annotate the SVs detected by Manta (Wang et al., 2010). Copy number variation (CNV) analysis was carried out by Control-FREEC software (Boeva et al., 2012). WGS data of cervical cancer tissue (Experiment ID: CRX040585) were used as an input. The ploidy parameter was set to 2 and other parameters were set as “default.”

### RNA-Sequence Analysis

RNA-Seq data of three normal (Experiment ID: CRX040582, CRX040583, and CRX040584) and two cervical cancer data (Experiment ID: CRX040580 and CRX040581) biological replicates were pre-processed as described (Andrews, 2010; Bolger et al., 2014) and mapped against Y-Chromosome less, HPV-16 and hg19 merged genome using HISAT2 tool (Kim et al., 2015). Gene expression abundance was quantified through featureCounts (Liao et al., 2014), and the gene expression level was calculated in RPKM value. Differentially expressed genes (DEGs) were detected by using DESeq2 R-package (Love et al., 2014). For enrichment analysis of DEGs, we used PANTHER online resource^4^ gene ontology (GO) tests, and statistical enrichment tests. To gain an overview of the gene pathway networks, web-based Kyoto Encyclopedia of Genes and Genomes (KEGG) server was recruited^5^. Furthermore, we used EnrichR (Chen et al., 2013) to assess the TF-lof enrichment, ENCODE TF ChIP-Seq enrichment, and Virus–Host Protein–Protein Interactions of selective genes list.

## Results

### Comparative 3D-Genome Structural Analysis

**FIGURE 1:**
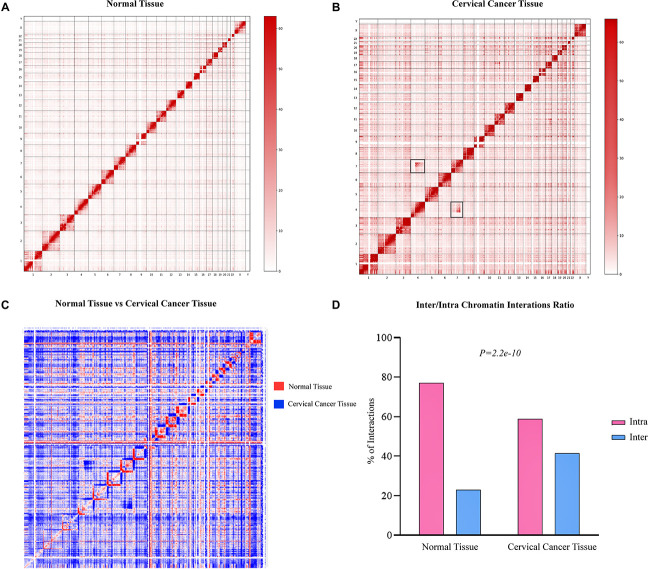
Hi-C data showing genome-wide variations in cervical cancer 3D-genome. Genome-wide Hi-C interaction map at 1 Mb resolution. Heatmap representing normal **(A)** and cervical cancer **(B)** tissues chromosomal interactions, respectively. Black squares indicate the inter-chromosomal rearrangements. **(C)** A heatmap shows the difference of higher order chromatin interactions between normal tissue (red color) and cervical cancer (blue color) tissue Hi-C data. **(D)** Hi-C quality analysis indicating the number of inter- and intra-chromosomal interactions between both samples. Red bars depict intra-chromosomal interactions, while sky-blue bars denote inter-chromosomal interactions.

**FIGURE 2:**
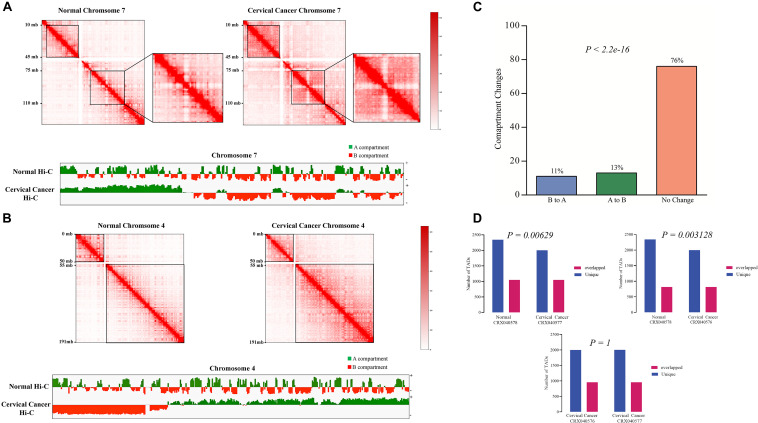
Comparative Hi-C data revealing the genome architecture changes at chromosomal level. Differential chromosomal architecture. **(A,B)** Comparison of chromatin interaction heatmaps from chromosomes 7 and 4 between normal (left panel) and cervical cancer (right panel) tissues. Variable regions are highlighted with the black boxes. Interaction maps are shown at 500 kb resolution. A/B compartment switching between normal and cervical cancer tissue was detected at 500 kb resolution using juicer-eigenvector. Values greater than 0 were assigned as A compartment (green color), and values less than 0 were designated as B compartment (Red color). **(C)** A/B compartment changes between normal and cervical cancer tissue data. Bar-graph representing overall compartment switching, orange: conserved compartments, green: A to B compartment conversion, and sky blue: B to A compartment change. **(D)** Bar-graphs show the comparison of the number of TADs found in cervical cancer and normal Hi-C data. Purple: TADs found in normal tissues, red: cervical cancer data in CRX040576 experiment, and blue: TADs found in cervical cancer tissue CRX040577 experiment.

In order to find the genome-wide structural architecture variations, we compared the cervical cancer and normal tissue Hi-C data. Four replicates of two cervical cancer experiments (Experiment ID: CRX040576 and CRX040577) and one replicate of normal tissue DLO Hi-C data (Experiment ID: CRX040578) were used for analysis (Supplementary Table 1). DLO Hi-C tool first filtered out the same (AA, BB) as well as the different (AB, BA) linkers, ∼285 million for normal tissues ∼146, ∼142, ∼156, and ∼162 million reads were obtained from four cervical cancer tissue replicates (Supplementary Table 2). Hi-C results showed the numbers of valid reads of normal sample CRX040578, cervical cancer tissue CRX040576, and cervical cancer tissue CRX040577 as 60,929,741, 28,518,853, and 36,389,304, respectively (Supplementary Table 3). Next, we visualized the whole-genome interaction map of normal and cervical cancer tissues to detect the differential arrangements. The higher order genomic organization was observed; apparently, the chromosomal architecture was consistent between normal and cervical cancer tissue heatmaps, but some chromosomes showed differential organizations (Figures 1A,B; Cao et al., 2020). We visualized the whole-genome contact matrices for both samples through juicebox (Durand et al., 2016a) and found that various regions showed the differential interactions frequency between different chromosomes (Figure 1C). The *cis-*interaction ratio between both samples was very similar, but the trans-interactions showed a significant increase (Fisher’s exact test *p*-value = 2.2e-10) (Supplementary Table 3 and Figure 1D). Several differential genomic organizations were detected in different chromosomes (Supplementary Figure 1). For example, in chromosome 7, higher order rearrangements were observed, and variable regions were found at 10–45 and 75–110 Mb regions (Figure 2A). Another variable region was present at 75–110 Mb position. A distinctive interaction pattern (enlarged and highlighted with the black square) appeared in cervical cancer tissues, but it was missing at the normal sample’s corresponding chromosomal region. In chromosome 4, two significant arrangements were observed from 0–50 and 55–191 Mb (highlighted with the black squares). The more dense architecture was observed at 55–191 Mb region in chromosome 4 of the cervical cancer sample (Figure 2B).

Further, we also checked the A/B compartments in both samples at 500 kb resolution. 76% of the total genome remained conserved, and only 13 and 11% of the genome showed compartment switching from A to B and B to A, respectively (Fisher’s exact test *p*-value < 2.2e-16) (Figure 2C). Moreover, we also monitored the A/B compartment switching at a chromosomal level such as in chromosome 7, 4 (Figures 2A,B), 1, and 16 (Supplementary Figure 1) several regions showed A/B compartment switching. It has been demonstrated that tumor development is associated with the alterations of TADs (Valton and Dekker, 2016). TADs at 40 kb resolutions were detected in each Hi-C experiment data; a total of 6,468, 6,033, and 6,268 TADs were found in normal tissue (CRX040578), cervical cancer tissue 1 (CRX040576), and cervical cancer tissue 2 (CRX040577), respectively. The comparison of TAD boundaries between experimental samples was calculated with *bedtools intersect -f 0.70* –r parameters and represented in the bar-graph (Figure 2D). We identified that 2,260 TADs were shared between all samples, 817 TADs were conserved, 1,998 and 2,342 were unique between cervical cancer tissue 1 (CRX040576) and normal tissue (CRX040578), respectively (Fisher’s test *p*-value = 0.00629), and 1,049 TADs found overlapped, 2,001 and 2,343 TADs were found as unique between cervical cancer tissue 2 (CRX040577) and normal tissues (CRX040578), respectively (Fisher’s test *p*-value = 0.003128). We also detected and compared the number of TADs between both cervical cancer samples (CRX040577 and CRX040576) and found 958 overlapped and 2,001 and 1,998 unique TADs which were statistically non-significant (Fisher’s test *p*-value = 1). Collectively, these results suggested that 3D-genome architecture shows differential behavior from normal to a cancerous condition.

### Translocation Identification in Cervical Cancer Hi-C Data

**FIGURE 3:**
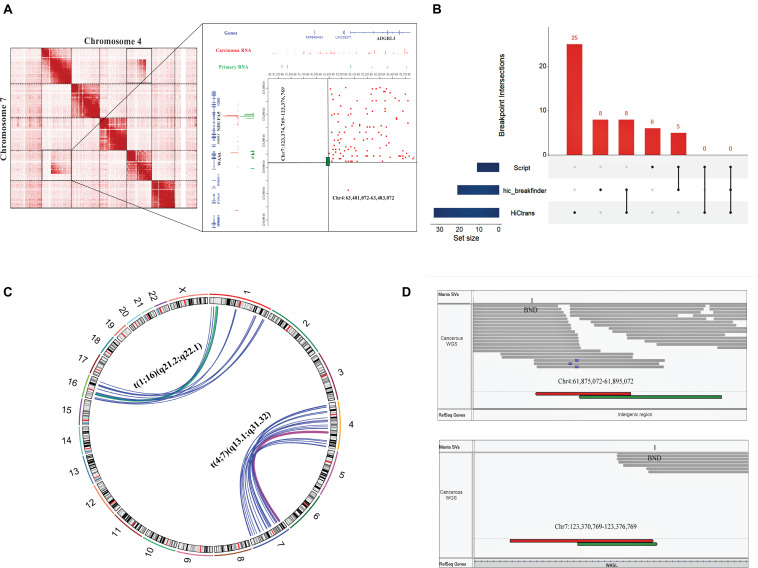
Hi-C and WGS results showing the consistency of translocations detected in cervical cancer samples. **(A)** Magnified representation of translocated regions observed by visual observation (left panel), while the right panel (zoomed snapshot) represents the Hi-C breakpoints between chromosome 7 and chromosome 4 detected by *in-house script* at highest resolution. Red peaks show cervical cancer RNA, while green color indicates the normal RNA sequence. Reference genes are highlighted with black color. **(B)** UpSet plot of breakpoints results detected by different methods. In the bottom left panel, blue horizontal bars represent the number of translocations detected by each method; vertical red-colored bars represent the size of breakpoint intersections of each translocation sets. Black dots show the sample set, and the intersection between methods is represented by a vertical black line. This graph is generated by UpSetR package. **(C)** Circos plot representing the chromosomal rearrangements between chr1, chr4, chr7, and chr16 detected by Hi-C and WGS. Deep pink- and green-colored arcs show the overlapped results of Hi-C and WGS, while blue color represents WGS-based translocations. **(D)** The translocation event between *t*(4;7) (q13.1; q31.32) in cervical cancer data visualized by IGV tool, showing WGS paired reads supporting the translocation results of Hi-C data. Breakpoints are labeled by “BND” in the upper panels of both IGV windows. Upper IGV window represents Chr4 reads, and the lower window shows Chr7 reads. Green and red colors depict the first and second read of mate-pairs.

We observed a large inter-chromosomal interaction region during Hi-C data analysis that suggests a translocation event in the cervical cancer sample (Experiment ID: CRX040576 and CRX040577). To predict the translocated area in a cervical cancer tissue sample, we further analyzed the Hi-C data through available tools such as hic_breakfinder (Dixon et al., 2018) and HiC-trans (Chakraborty and Ay, 2018). hic_breakfinder uses bam input and detects translocations by creating sub-matrices of the original matrix that potentially contains chromosomal rearrangements (Dixon et al., 2018). It predicted seven breakpoints in cervical cancer sample 1 (CRR045289) and 3 (CRR045291) each, and eight breakpoints in cervical cancer sample 2 (CRR045290) and sample 4 (CRR045288). A total of seven chromosomal pairs were observed that undergo translocations, such as chr2-chr12, chr3-chr12, chr4-chr7, chr16-chr1, chr6-chr5, chr17-chr11, and chr3-chr6; the breakpoint boundaries for each pair are given in Supplementary Table 4. HiC-trans detected several chromosomal pairs with the translocations at different bin sizes 40, 80, and 120 kb; in cervical cancer Hi-C sample 1 (CRR045289) 25, in cervical cancer Hi-C sample 2 (CRR045290) 35, in cervical cancer Hi-C sample 3 (CRR045291) 49, and in cervical cancer Hi-C sample 4 (CRR045288) it predicted 54 breakpoints (Supplementary Table 5). We observed that hic_breakfinder and HiC-trans use different detection approaches by considering different biases that resulted in more false positive detections. To overcome that issue, we build an *In-house script* that detects the obvious breakpoints using interacting pair files as input. It predicted six translocated chromosomal pairs in cervical cancer Hi-C sample 1 (CRR045289) in such a way that two in chr4-chr7 and four breakpoints in chr1-chr16 pair that show translocations (Supplementary Figure 2). In other cervical cancer Hi-C samples (CRR045290, CRR045291, and CRR045288), our *script* predicted two breakpoints in chr4-chr7 pair each (Supplementary Table 6). The translocation between chr7:123,374,769–123,376,789 and chr4:63,481,072–63,483,072 is shown in Figure 3A. The translocated region between chr1: 144,816,374–144,826,374 and chr16:70,838,537–70,848,537 is shown in Supplementary Figure 2.

We compared the translocation boundaries detected by publically available tools with our *script*; we used *bedtools pairToPair* to find the overlaps and unique translocations. Here, we took one sample as an example in which hic_breakfinder detected eight breakpoints, HiCtrans 25, and our *script* detected six breakpoints. After comparison, we found that eight breakpoint regions were overlapped between hic_breakfinder and HiCtrans. Five breakpoint regions were similar between the results of *in-house script* and hic_breakfinder. Neither had we detected any overlap between HiCtrans and our *script* nor in all other tools (Figure 3B).

### Structural Variation Detection in Whole-Genome Sequence Data

We analyzed WGS data of the corresponding cervical cancer tissue data of patients (Experiment ID: CRX040585) to find the novel SVs such as deletions, translocations, insertions, and duplications, and to evaluate the precision of translocations detected in Hi-C data. Sequence quality was determined by FastQC (Andrews, 2010). Sequence contaminations such as overrepresented sequences and low-quality reads were clipped using appropriate sequence clipper in Trimmomatic (Bolger et al., 2014). Total 99.66% reads were used for mapping, and 97.82% read pairs were adequately aligned against the customized reference genome (hg19 + hpv16). The average read depth of the cervical cancer sample was calculated by SAMtools (Li et al., 2009), which was ∼45X. Manta Tumor-Only Analysis was performed to find the structural variants (Chen et al., 2016). Manta predicted that 3,579 reads have maximum depth, 301 reads did not match with default filtration score or aligned to multiple locations around the breakpoints, and 16,712 variants passed the filtration threshold score of Manta.

Further, we split the genome-wide SVs into their respective types, such as 7,858 inter-chromosomal translocations breakpoints (BND), 5,799 deletions (DEL), 1,185 duplications (DUP), and 1,870 inversions (INV). Our primary focus was to check the consistency of translocation results of Hi-C data, such as chr4-chr7 and chr1-chr16, with variations identified by the Manta tool. We found that the positions of chr7-chr4 and chr1-chr16 breakpoints were coherent with inter-chromosomal translocation identified from WGS data (Figure 3C). Additionally, we have also inspected the WGS paired read analysis, and found the presence of translocated mate-pair reads in chr1-chr16 (Supplementary Figure 2) and chr4-chr7 (Figure 3D). Moreover, we detected the protein-coding genes at the translocated regions, specifically in chr4:61,875,072–61,895,072 and chr7:123,370,769–123,376,769 and chr1:144,816,374–144,826,374 and chr16:70,838,537–70,848,537 region. WASL gene was found at chromosome 7 (q31.3) (Figure 3A), NBPF20 at 1 (q21.1), and HYDIN gene was present at chromosome 16 (q22.2) (Table 1 and Supplementary Figure 2). WGS annotation results suggested that translocation between chr4-chr7 has a very “high” impact on this WASL gene. Copy number variation is another key phenomenon that contributes to cancer development. So, Control-FREEC identified several copy number variations (CNVs) in multiple chromosomes such as chromosome 1, 2, 4, 8–11, 16, 18, and 21. A total 249 “gain” and 32 “loss” events occurred (Supplementary Figure 3 and Supplementary Table 7). These results collectively showed that translocations identified by Hi-C data are consistent with the WGS data. Additionally, we found several protein-coding genes at the translocated region directly involved in female cancer development.

**TABLE 1:** Translocation breakpoints and neighboring genes around translocated pairs.

Karyotype	Breakpoint coordinates	Disrupted genes	Neighboring genes
*t*(4;7) (q13.1; q31.32)	Chr4:61,875,072–61,895,072 Chr7:123,370,769–123,376,769	Intergenic region WASL	ADGRL3 NDUFA5
*t*(1;16) (q21.2; q22.1)	Chr1:144,816,374–144,826,374 Chr16:70,838,537–70,848,537	NBPF20 HYDIN	VAC14 SF3B3 COG4 CALB2 ZNF23 ZNF19

### Effects of Translocations on Gene Expression

**FIGURE 4:**
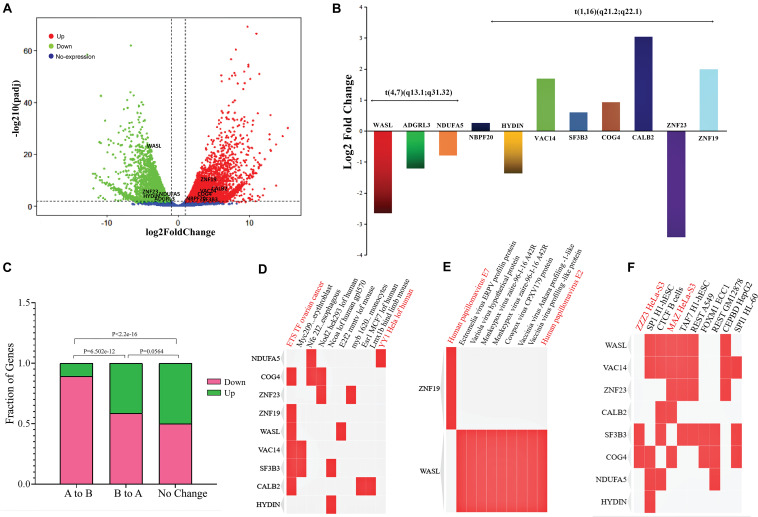
RNA-Seq data results revealing the expression changes due to A/B compartments switching and in genes present around the translocations sites. **(A)** Volcano plot showing differentially expressed genes (DEGs); red dots: upregulated genes, green dots: downregulated genes, and blue dots: genes with no expression changes. **(B)** Expression changes of the genes (log2FC) present around *t*(4,7) (q13.1; q31.32) and *t*(1;16) (q21.2; q22.1); each gene is represented with a different color and the height of a bar indicates the Log2 fold change value. **(C)** Bar-plot shows the effects of A/B compartment switching on change in gene expression; the height of a bar represents the fraction of DEGs; green color: upregulated genes while deep pink: downregulated genes. **(D–F)** Clustergram of TF-lof expression of GEO enrichment library, Virus–Host Protein–Protein Interaction enrichment, and ENCODE TF ChIP-seq libraries, respectively. Enrichment terms are shown in each column, each row indicates the candidate genes, and matrix shows the association between gene and types of enrichment term. Red color indicates the terms directly or indirectly associated with female cancers (cervical cancer/ovarian cancer).

Previously, it is reported that SVs play a significant role in changing gene expression, leading to cancer development. To explore the effect of SV mainly the translocations on the expression of surrounding genes, we used the transcriptome of cervical cancer and normal cervix tissue. A total of ∼8,000 genes were detected as DEGs (Supplementary Table 8) that fulfill the criteria of false discovery rate (FDR) < 0.05 and absolute Log2 fold change (Log2 FC) > 1 (Figure 4A). Next, we were curious to determine the effects of translocation on the expression of genes present within translocated regions and in their vicinity. In *t*(4;7) (q13.1; q31.32), WASL gene (7q31.3) underwent translocation, showed expression disruption, and was significantly downregulated, while at chromosome 4 (4q13.1), an intergenic region was observed. We further extended our analysis in the neighboring (within ± 1 Mb region) genes at chromosome 4, such as ADGRL3 and NDUFA5, and found that both genes were downregulated (Figure 4B). We also monitor the gene expression changes in *t*(1;16) (q21.2; q22.1) and nearby regions. This translocation occurred within NBPF20 and HYDIN genes located at 1q21.2 and 16q22.1, respectively (Supplementary Figure 2). The former was upregulated, and the latter was downregulated. The neighboring genes such as ZNF23 showed downregulated expression, while VAC14, SF3B3, COG4, CALB2, and ZNF19 appeared as upregulated genes (Figure 4B and Table 1).

Since our Hi-C results showed a significant A/B compartment switching, previous studies have already reported the correlation between compartments switching and gene expression changes (Wu et al., 2017). So, here we aimed to check how many genes were affected by compartment switching in translocated chromosomes (1, 4, 7, and 16). In A to B compartment change, a total of 239 and 36 genes were found as down- and upregulated, respectively, while in B to A, 69 genes were downregulated and 49 were detected as upregulated genes. In the no-change category, 688 genes showed upregualted and 691 showed downregulated expression. Fisher’s exact test suggested that gene expression changes between A to B and B to A category were fairly significant (Fisher’s exact test *p-value* = 6.502e-12). In B to A and no-change category, a significant (Fisher’s test *p-value* = 0.0564) number of genes were found to be changing gene expression, while the number of genes changing expression in A to B and conserved genome category was found as highly significant (Fisher’s exact test *p-value* < 2.2e-16) (Figure 4C).

In GO analysis, we predicted the overall enrichment of DEGs in cellular components and molecular functions. Results suggested that the DEGs were involved in maintaining different cellular components and regulating various molecular functions such as structural constituent of ribosome (18%), immune receptor activity (12%), cytokine binding (12%), structural molecular activity (8%), and signaling receptor binding (8%) (Supplementary Figure 4).

**FIGURE 5:**
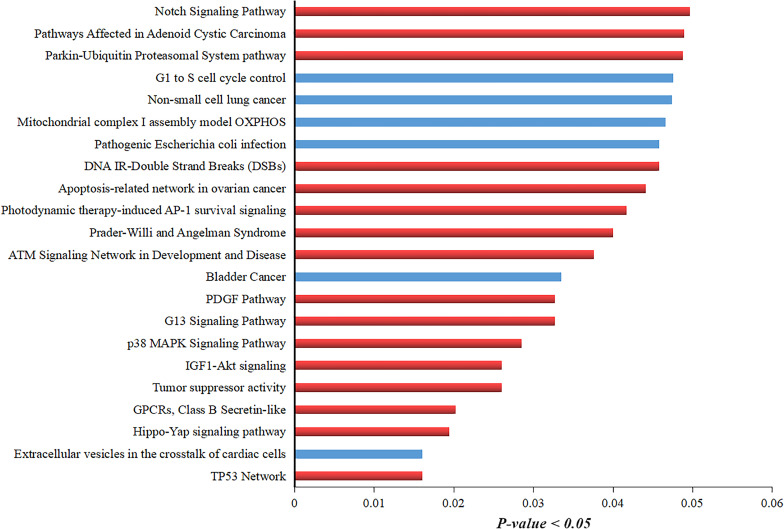
GO analysis of translocation neighboring genes showing their involvement in different pathways controlling cervical cancer development. Pathway enrichment analysis of translocations neighboring genes. Red-colored bar indicates the pathways that are directly involved in HPV-mediated cervical cancer development. Blue-colored bar shows the pathways involved in other types of cancers.

Additionally, we extracted the list of genes around the translocation and performed GO using TF-lof expression, ENCODE TF ChIP-seq, and Virus–Host PPI enrichment libraries by enrichR annotation platform. Results showed that in TF-lof expression library enrichment, all genes except ZNF23, NDUFA5, and HYDIN were controlled by previously reported *ETS* transcription factor (up expression) of ovarian cancer (Llauradõ et al., 2012). NDUFA5 gene was significantly enriched with the transcription factor *YY1* (down-expression) of HeLa cell line consistent with the previous studies of human (Rizkallah and Hurt, 2009). ZNF23 showed *myb* TF enrichment in primary monocytes of humans (Huang et al., 2007; Figure 4D). Virus–Host PPI enrichment analysis suggested that WASL and ZNF19 genes were highly enriched in interacting with HPV E7 and HPV type 144 proteins, respectively (Figure 4E; White et al., 2012). ENCODE TF ChIP-Seq data library enrichment results showed that SF3B3 and COG4 were enriched by transcription factor *ZZZ3* and WASL, ZNF23, VAC14, and CALB2 genes were controlled by *MAZ* TF of HeLa-S3 (cervical cancer) cell line (Figure 4F; Dolfini et al., 2016). Several other TFs were also detected that potentially influence the transcription of the genes mentioned above. Pathway enrichment analysis showed the association of these genes in carcinomal pathways such as TP53 pathway (Tommasino et al., 2003), tumor suppressor pathway (Cohen et al., 2003), PDGF pathway (Li et al., 2017), p38 MAPK signaling pathway (Kang and Lee, 2008), G13 signaling pathway (Yuan et al., 2016), and Notch signaling pathway (Rodrigues et al., 2019; Figure 5). Overall, we concluded that gene expression significantly changed around translocated region in cervical cancer samples and A/B compartment changes lead to change in gene expression; moreover, the enrichment analysis suggested that the regulation of genes present at translocation regions was controlled by previously reported transcription factors of cervical cancer/ovarian cancer studies. Additionally, we have also predicted the direct role of newly identified genes in cervical cancer-related pathways.

## Discussion

Cervical cancer is the leading type of female cancer caused by HPV genome integration (Li et al., 2021). HPV integration has complex effects on the phenotype, and the mechanism driving these effects is poorly understood. Some studies have reported that structural rearrangements are also the driving force behind tumorigenesis (Zhang et al., 2018). These SVs cause higher order disorganizations, which we assume play a significant role in changing gene expression and ultimately result in tumor development and progression around the cervix tissues. Several studies have shown that SVs, including duplications, deletions, translocations, insertions, and inversions, can disrupt the 3D-genome specifically the TAD boundaries, in a way that they can produce neo-TADs, fused-TADs, or can cause the deletion of TADs (Valton and Dekker, 2016). In HPV-induced cervical cancer, the remodeling of TADs is associated with the enhancer-hijacking (Cao et al., 2020), which leads to the change in gene expression followed by the deleterious phenotypes such as developmental disorders and cancer (Melo et al., 2020). Moody and Laimins (2010) have reported that in HPV + state, viral E6 and E7 oncogenes are predominantly attributable to the SVs/instability of the host genome. Previous research has reported that HPV-integrations sites are prone to change in the local structure of host loci and gene expression (Cao et al., 2020). The detection of SVs is still a burning issue in cancer research. This study used Hi-C, RNA-Seq, and WGS data of cervical patients to perform a comparative analysis between cervical cancer and normal tissue. Here, we identified the genome-wide SVs, specifically the translocations away from the HPV-integration point using Hi-C-based translocation detection methods, and to monitor their effects on gene expression around the breakpoints. Hi-C data from four cervical cancer patients were used to detect the chromosomal interactions and rearrangements; comparative results showed that overall 3D-genome architecture appeared to be consistent between normal and cervical cancer tissue data consistent with the findings of the previous study (Cao et al., 2020), although some chromosomes showed higher order chromatin structure variations, including a significant change in the number of TADs and A/B compartment switching.

Furthermore, we have observed some translocations in the genome-wide interaction map. HiC-Trans (Chakraborty and Ay, 2018) and hic_breakfinder (Dixon et al., 2018) were used to locate the translocations precisely. Both tools produced a higher number of breakpoints, and a higher number suggested the high false discovery rate (FDR) (Wang et al., 2020). To overcome this problem, we have designed *in-house script* that only detected the obvious translocation breakpoints at the highest resolution and less computational cost. Two translocations such as *t*(4;7) (q13.1; q31.32) and *t*(1;16) (q21.2; q22.1) were detected. WGS data validated the presence of translocations detected by the *in-house script*.

Additionally, WGS paired read analysis found the presence of translocated reads in chr1-chr16 (Supplementary Figure 2) and chr4-chr7. WGS results were coherent with the breakpoints detected by the newly designed script in Hi-C data. The coherence of Hi-C and WGS results confirmed the sensitivity and specificity of our *in-house script* output. Further, annotation results suggested the presence of intergenic regions and coding genes such as WASL, NBPF20, and HYDIN at the breakpoint region. Previous studies have suggested the active role of NBPF20 in gene fusion in cervical cancer patients (Li et al., 2021). Mutagenesis studies of gynecological cancers have revealed that HYDIN gene undergoes frequent mutations in both ovarian and cervical cancer (Guo et al., 2020). Published research has demonstrated that the WASL (Wiskott–Aldrich Syndrome-Like) gene belongs to the oncogenes category and plays a significant role in tumor progression and metastasis in cervical cancer (Hidalgo-Sastre et al., 2020). CNV analysis depicted that chromosomes 1, 4, 7, and 16 were among the chromosomes which undergo copy number variations consistent with the previous cervical cancer studies (Adey et al., 2013). Collectively, 3D-genome structure, WGS, and CNVs results showed a strong association between the chromosomal architecture and breakpoints.

Chromosomal translocations lead to the disruption of gene expression and cause proto-oncogenes activation (Rabbitts, 1994). Some studies also reported that SVs in general have multiple local and global effects on chromosomal structure, chromatin interactions, and gene expression (Zhang et al., 2018). Here, we aimed to study the potential impact of translocations on gene expression. The transcriptome data of cervical cancer suggested many upregulated and downregulated genes compared to the normal tissue sample. We obtained the neighboring genes around the breakpoints regions in chromosomes 1, 16, 4, and 7 and found the disrupted gene expression. GO analysis depicted that DEGs were involved in various molecular functions and cellular processes.

Furthermore, we performed various enrichment analyses using different GO libraries such as TF-lof expression of GEO enrichment, Virus–Host Protein–Protein Interaction enrichment, and ENCODE TF ChIP-seq enrichment libraries. TF-lof expression of GEO enrichment results showed that the most of the detected genes were enriched with the previously reported transcription factors YY1 of Hela-cells (cervical-cancer cell line) (Rizkallah and Hurt, 2009) and ETS transcription factor of ovarian cancer (Llauradõ et al., 2012). Virus–Host PPI enrichment results suggested the interaction enrichment of disrupted genes such as ZNF19 with HPV-E7 and WASL with HPV-E2 (Cohen et al., 2003). Next, we examined the regulatory network of detected genes through ENCODE TF ChIP-Seq enrichment library and found that these genes were enriched with the previously reported TFs of Hela-S3 (cervical cancer) cell lines.

Pathway analysis results also showed that the genes located in breakpoint regions are strongly associated with various cancer-mediated pathways such as TP53 network, GPCRs Class B Secretin-like pathway, p38 MAPK signaling pathway, apoptosis-related network in ovarian cancer, Notch signaling pathway, PDGF pathway, and IGF1-Akt signaling (Steller et al., 1996; Serrano et al., 2008; Chen, 2015; Wu et al., 2017). Overall, these findings provide strong evidence that breakpoints occurred in the genes that have a strong correlation with HPV-mediated cervical cancer.

Although we have predicted some novel translocations, there are some limitations associated with this study, for example, the limited availability of a dataset used to carry out the analyses. Increasing the size of cohort will help to get better understandings about cervical cancer development mechanism. Another major limitation is the heterogeneous nature of cervical cancer making the study more challenging. Combining all analyses, we unveil that in cervical cancer, multiple genomic alternations such as translocations, CNVs, and 3D reorganization occur that affect the gene expression.

These findings shed light on the importance of studying the effects of SVs on the 3D genome and finding candidate genes in cervical cancer. We believe that this will help us to improve our understandings of the HPV-mediated cervical cancer mechanism and identify the targeted clinical therapeutics against cervical cancer.

## Data Availability Statement

The datasets presented in this study can be found in online repositories. The names of the repository/repositories and accession number(s) can be found in the article/Supplementary Material. The *in-house script* is available at https://github.com/Adeel3Dgenomics/In-house-script-for-translocation-detection.

## Author Contributions

MA and GL: conceptualization, writing—original draft preparation, and writing—review and editing. MA: design. HJ: software. CC: sample collection. DL and CC: biological experiments. MA, HJ, KC, and YA: formal data analysis. GL, PW, and GC: supervision. GL and PW: funding acquisition. All authors contributed to the article and approved the submitted version.

## Conflict of Interest

The authors declare that the research was conducted in the absence of any commercial or financial relationships that could be construed as a potential conflict of interest.

## Footnotes

1 https://bigd.big.ac.cn/gsa/s/P37lFNi0

2 https://github.com/dixonlab/hic_breakfinder

3 http://broadinstitute.github.io/picard/

4 www.pantherdb.org

5 http://www.genome.jp/kegg/kaas/

## Supplementary Material

Supplementary Figure 1 Hi-C results showing the comparative analysis for chromosome 1 and chromosome 16 in both normal and cervical cancer samples. Differential chromosomal architecture: **(A)** Chromosome 1 of normal tissue (left) and cervical cancer tissue (right) Hi-C heatmaps. A/B compartments between both samples have been shown in the lower panel of A. **(B)** Chromosome 16 of the normal sample (left) and cervical cancer tissue (right). A/B compartments are shown in the bottom panel. Dark Green; A compartment, Red; B compartment.

Supplementary Figure 2 Hi-C-based translocation detected in chromosome 1 and 16 was coherent with the WGS mate-pairs analysis. **(A)** Enlarged Hi-C interaction map shows the breakpoint position corresponding to translocation *t*(1;16) (q21.2; q22.1). NBPF20 and HYDIN genes were found at the translocation region. RNA-seq peaks are also visualized, red-colored peaks indicate cervical cancer RNA, and green color shows normal sample gene expression. **(B)** Visualization of translocated read pairs in translocation event *t*(1;16) (q21.2; q22.1). Red: translocated read mate-pairs.

Supplementary Figure 3 Copy number variations detected in cervical cancer sample showing that translocations containing chromosomes also undergo CNVs. Estimated genome-wide copy number variations (CNVs) from cervical cancer whole-genome sequence (WGS) data. Red dots represent “gains,” blue dots show “loss” events, and green dots show “normal” events.

Supplementary Figure 4 Gene ontology analysis of DEGs. Enrichment of genes in various **(A)** cellular components; red bars indicate the cellular component term and length represents the −log10 (FDR). **(B)** Molecular functions; each slice of the circle depicts the molecular function and gene involvement percentage.

Supplementary Table 1 Data source for cervical cancer. The table shows the experiment ID, sequencing run accession number, sequence type, and total reads.

Supplementary Table 2 Linkers filtering information. Linkers detected by DLO Hi-C tool from normal tissue and cervical cancer tissues (1, 2, 3, and 4) DLO Hi-C dataset.

Supplementary Table 3 Hi-C data analysis stats for normal and cervical cancer samples. Hi-C results for each experiment; each table shows the information of self-ligated and non-ligated pairs, valid reads, intra–inter chromosomal interactions, and long- and short-range chromatin interactions.

Supplementary Table 4 Translocations detected by hic_breakfinder. Translocations detected by hic_breakfinder in cervical cancer samples, the table showing the translocation boundaries along with the strand information.

Supplementary Table 5 Translocations identified by HiC_Trans. Translocations detected by HiC_Trans in cervical cancer samples, the table showing the breakpoint boundaries along with the z-score.

Supplementary Table 6 Translocations identified by *in-house* script. Breakpoints detected by in-house build script in cervical cancer sample Hi-C data, the table showing the breakpoint boundaries information of translocated regions.

Supplementary Table 7 Copy number variations (CNVs) in cervical cancer WGS. CNVs identified by Control-FREEC in cervical cancer data, number of gains, and loss events are shown.

Supplementary Table 8 Gene expression data for cervical cancer tissue. DEGs detected in cervical cancer and normal RNA-seq data of cervical cancer patients.
