# Supplementary figures and images for: Structural Variations of the 3D Genome Architecture in Cervical Cancer Development
Source: Front Cell Dev Biol. 2021 Jul 23;9:706375. doi: 10.3389/fcell.2021.706375 (PMC8344058; doi:10.3389/fcell.2021.706375)

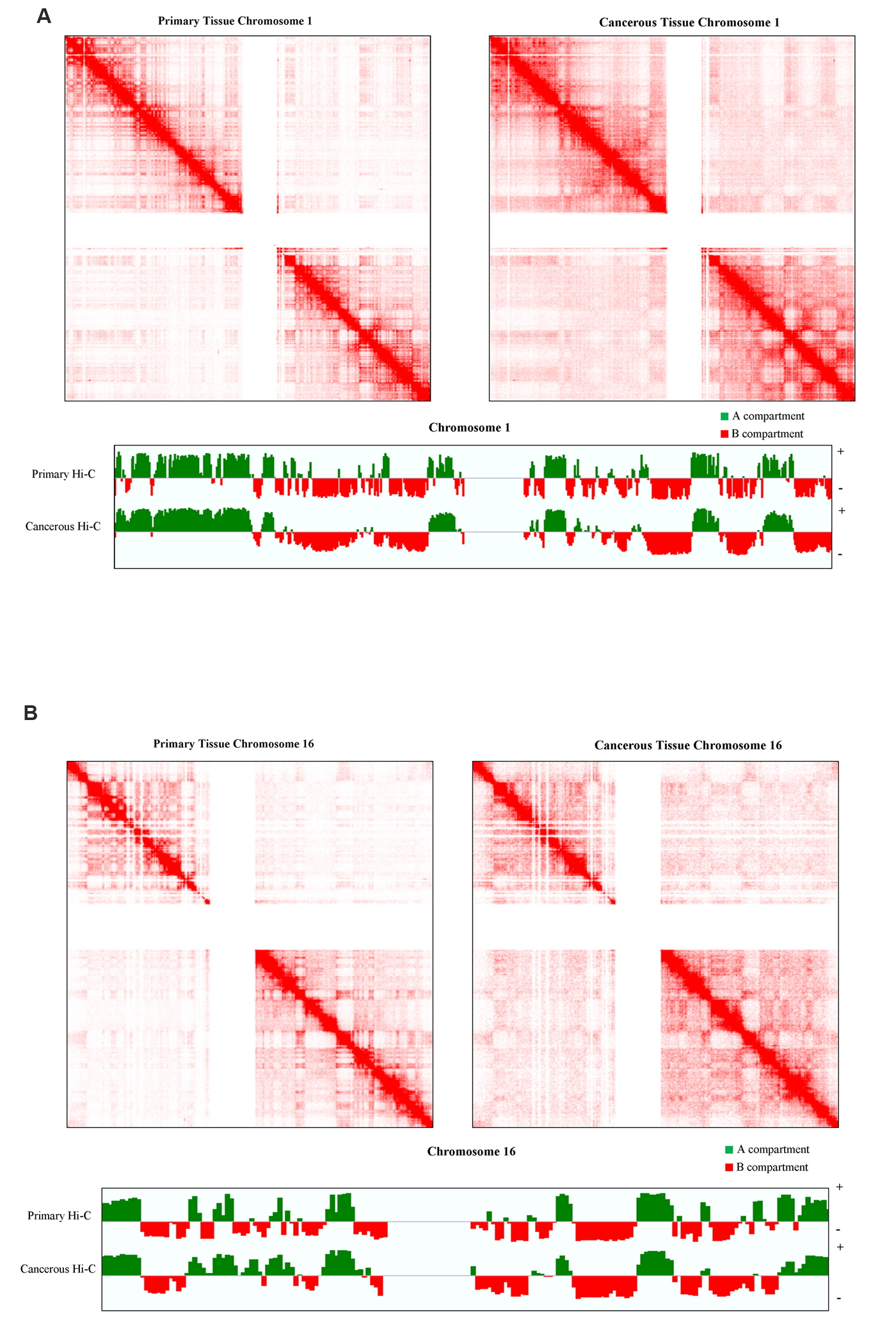

Supplement: Supplementary Figure 1 — Hi-C results showing the comparative analysis for chromosome 1 and chromosome 16 in both normal and cervical cancer samples. Differential chromosomal architecture: (A) Chromosome 1 of normal tissue (left) and cervical cancer tissue (right) Hi-C heatmaps. A/B compartments between both samples have been shown in the lower panel of A. (B) Chromosome 16 of the normal sample (left) and cervical cancer tissue (right). A/B compartments are shown in the bottom panel. Dark Green; A compartment, Red; B compartment. [file Image_1.TIF]

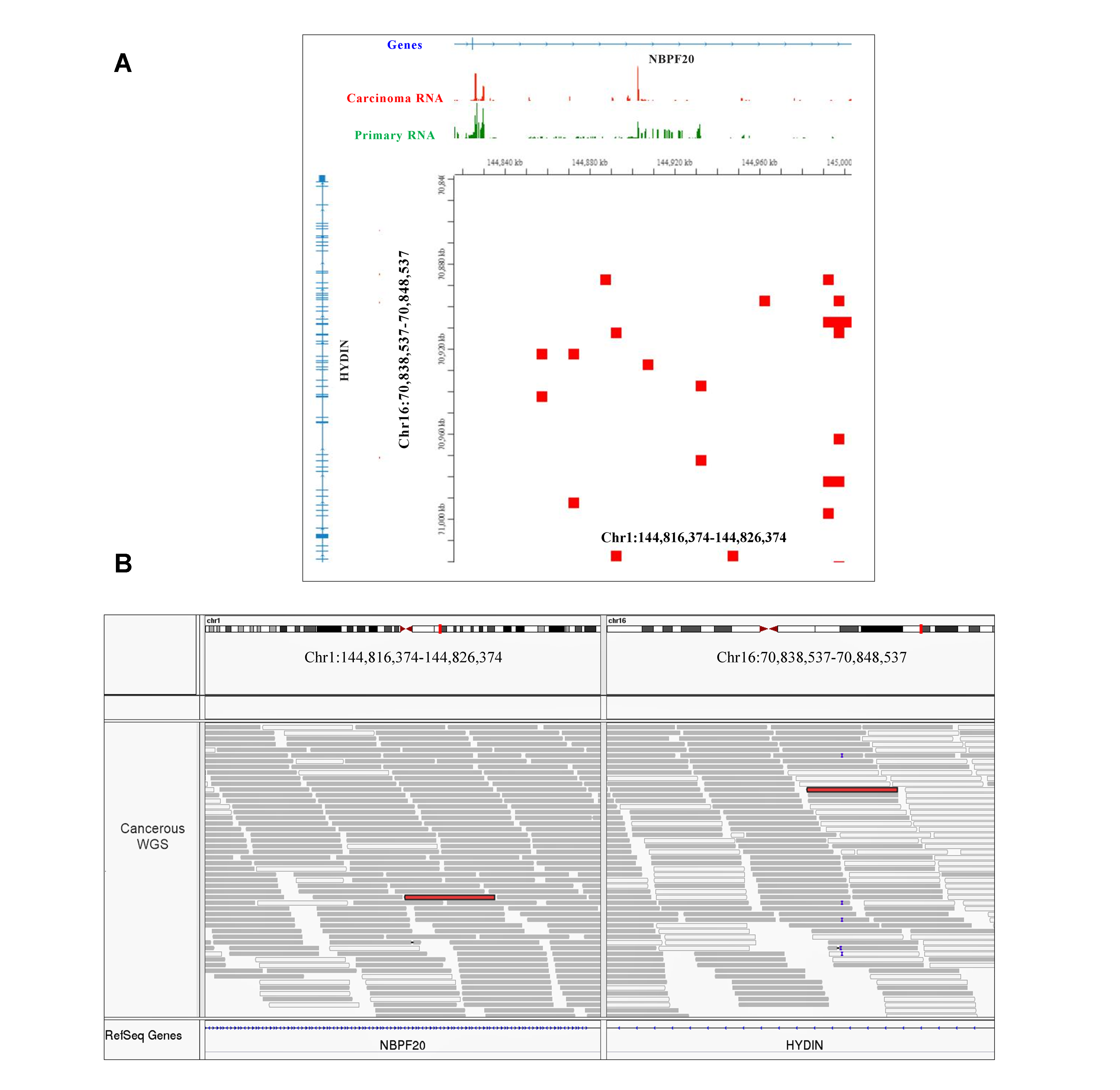

Supplement: Supplementary Figure 2 — Hi-C-based translocation detected in chromosome 1 and 16 was coherent with the WGS mate-pairs analysis. (A) Enlarged Hi-C interaction map shows the breakpoint position corresponding to translocation t(1;16) (q21.2; q22.1). NBPF20 and HYDIN genes were found at the translocation region. RNA-seq peaks are also visualized, red-colored peaks indicate cervical cancer RNA, and green color shows normal sample gene expression. (B) Visualization of translocated read pairs in translocation event t(1;16) (q21.2; q22.1). Red: translocated read mate-pairs. [file Image_2.TIF]

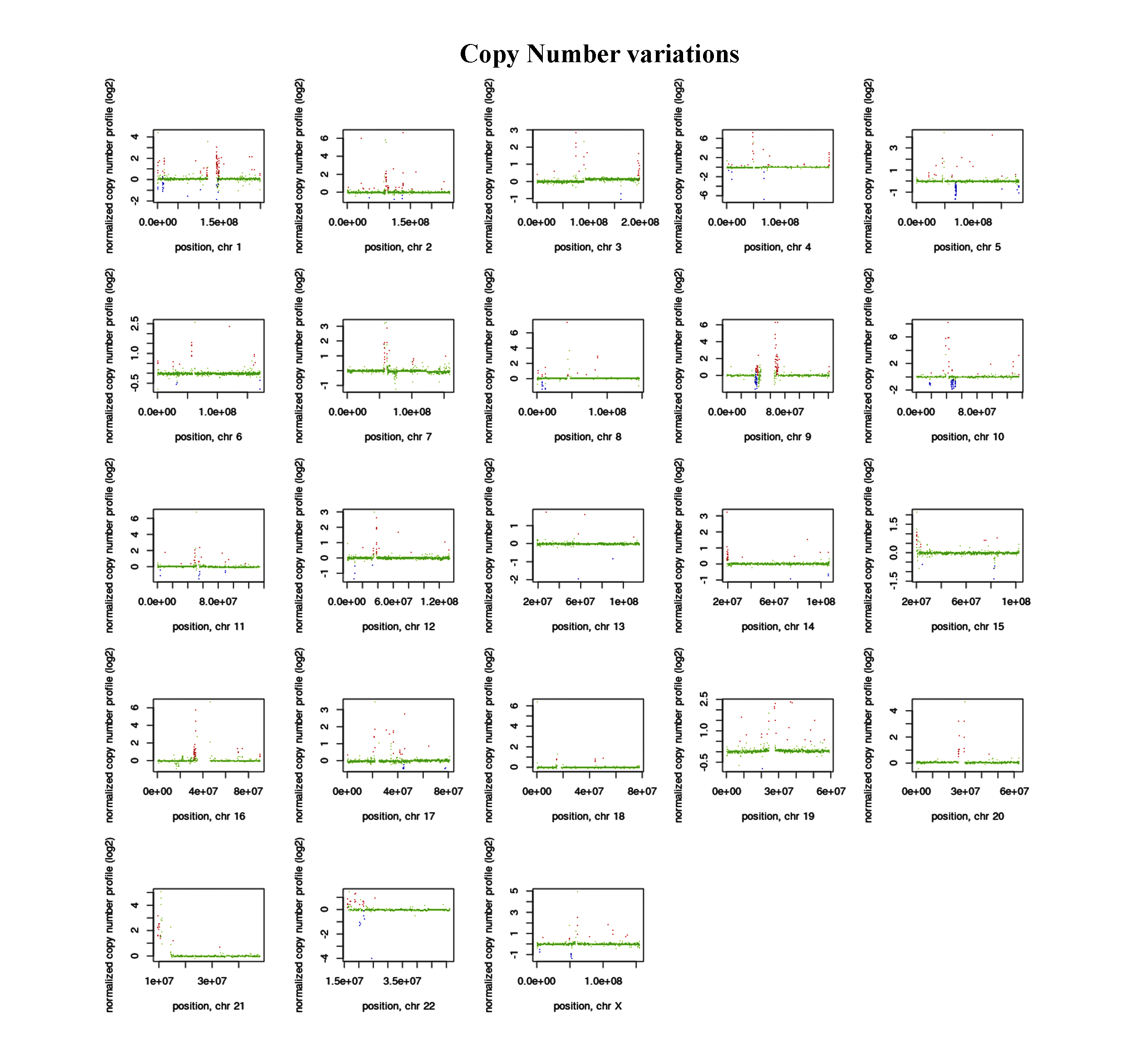

Supplement: Supplementary Figure 3 — Copy number variations detected in cervical cancer sample showing that translocations containing chromosomes also undergo CNVs. Estimated genome-wide copy number variations (CNVs) from cervical cancer whole-genome sequence (WGS) data. Red dots represent “gains,” blue dots show “loss” events, and green dots show “normal” events. [file Image_3.TIF]

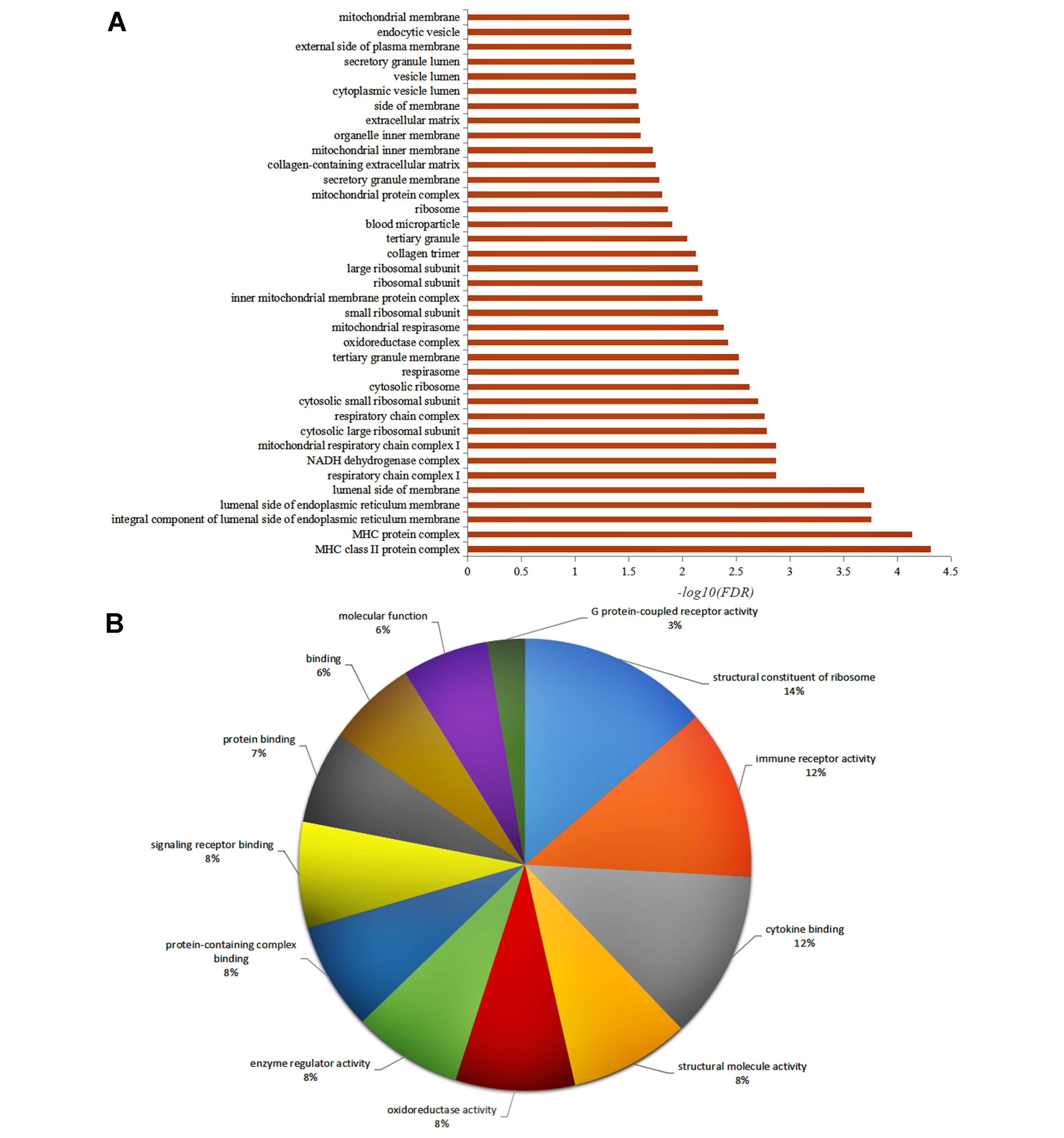

Supplement: Supplementary Figure 4 — Gene ontology analysis of DEGs. Enrichment of genes in various (A) cellular components; red bars indicate the cellular component term and length represents the −log10 (FDR). (B) Molecular functions; each slice of the circle depicts the molecular function and gene involvement percentage. [file Image_4.TIF]
